# Matrix metalloproteinase-13 refines pathological staging of precancerous colorectal lesions

**DOI:** 10.18632/oncotarget.12429

**Published:** 2016-10-04

**Authors:** Anna-Katharina Wernicke, Yuri Churin, Diana Sheridan, Anita Windhorst, Annette Tschuschner, Stefan Gattenlöhner, Martin Roderfeld, Elke Roeb

**Affiliations:** ^1^ Department of Gastroenterology, Justus-Liebig-University, Giessen, Germany; ^2^ Department of Pathology, Justus-Liebig-University, Giessen, Germany; ^3^ Institute for Medical Informatics, Justus-Liebig-University, Giessen, Germany

**Keywords:** MMP, neoplasia, colon cancer, adenoma, immunoreactive scoring

## Abstract

An exact classification of precancerous stages of colorectal polyps might improve therapy and patients' outcome. Here we investigate the association between grade of dysplasia and Matrix metalloproteinase-13 (MMP-13) expression in 137 biopsies from patients with cancerous and non-cancerous colorectal adenomas. A reproducible staining procedure for histologic MMP-13 analysis in routinely fixed colorectal biopsy specimens has been established. A newly adopted immunoreactive scoring system for MMP-13 was demonstrated as reliable readout.

The strength of the association between pathologic stage and immunoreactive MMP-13 scoring emphasizes its eligibility for diagnosis in precancerous colorectal lesions.

## INTRODUCTION

Colorectal cancer (CRC) is the third most frequently diagnosed cancer in the world [1]. Based on 18 studies, the pooled prevalence of adenomas, colorectal cancer, non-advanced adenomas, and advanced adenomas was 30.2%, 0.3%, 17.7%, and 5.7%, respectively [2]. In a population-based case-control study, the risk of CRC was strongly reduced after colonoscopy for any indication [3]. A key point for improving the survival rate is the correct diagnosis and treatment at an early stage [4].

Most cases of colorectal carcinogenesis are characterized by enhanced expression of MMP-3, −7, and −13 [5]. MMP-7 and MMP-13, which are expressed primarily on the tumor cell surface, are elevated in inflammatory bowel disease [6]. Studies indicate that expression of MMP-13 is closely related to the development and progression of colorectal cancer [7–9]. Immunoreactivity to MMP-13 was identified in 91% and localized to tumor cells. A high MMP-13 staining score showed a trend towards poorer survival [7] and a high MMP-13 expression level is associated with a high rate of liver metastasis [9]. Furthermore, positive MMP-13 expression was related to bad prognosis and early relapse [7, 10]. Thus, MMP-13 could be a useful indicator for tumor behavior and prognosis of CRC.

Our aim was to determine whether MMP-13 expression in colorectal adenomas and carcinomas is useful for a concise and accurate diagnosis. Therefore we assessed the association between grade of dysplasia and MMP-13-expression. The characterization of the CRC-specific biomarker MMP-13 should facilitate objective and early detection of high grade adenomas and carcinomas.

## RESULTS

MMP-13 expression was assessed in 137 biopsy samples from 105 patients with colorectal adenomas and colorectal cancer (Table 1).

**Table 1:** Demographic and clinical data of the cohort, n = 105

Demographics	*n*	percentage/ total
Women	34	32.4%
Men	71	67.6%
**Age**		
Mean	66.7	
SD	13.4	
**Histological Controls**		
Tumor free resection margins	15	
**Histiologic type**		
tubular	37	30.3%
tubulovillous	70	57.4%
hyperplastic	15	12.3%
**Dysplasia**		
hyperplastic	15	12.3%
lowgrade	64	52.5%
highgrade	20	16.4%
carcinoma	23	18.9%
**Localisation**		
Rectum	25	20.5%
Rektosigmoid	5	4.1%
Sigmoid Colon	28	23.0%
Descending Colon	10	8.2%
Left colic flexure	0	0.0%
Transverse Colon	8	6.6%
Right colic flexure	10	8.2%
Ascendic Colon	23	18.9%
Cecum	13	10.7%

Mean age was 67 ± 13 years and 34 (32.4%) patients were female. Samples included 15 (12.3%) hyperplastic adenomas, 64 (52.5%) low grade adenomas, 20 (16.4%) high grade adenomas and 23 (18.9%) carcinomas. Following histological types were found: 15 (12.3%) were hyperplastic, 37 (30.3%) were tubular and 70 (57.4%) tubulovillous.

**Figure 1:**
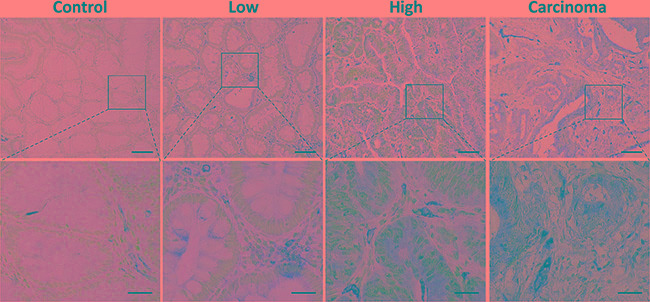
Increasing expression of MMP-13 in colonic adenoma-carcinoma sequence Immunohistochemical staining for MMP-13 (brown) in low and high grade adenoma, healthy controls, and carcinoma demonstrates a correlation between neoplastic grade and MMP-13 expression. Representative micrographs are shown. Nuclei are stained blue. Scale bars: 100 μm, upper panels 100× and 25 μm, lower panels 400×.

The MMP-13 expression increased with pathologic stage of the (pre-) cancerous mucosal lesions (Figure 1).

**Figure 2:**
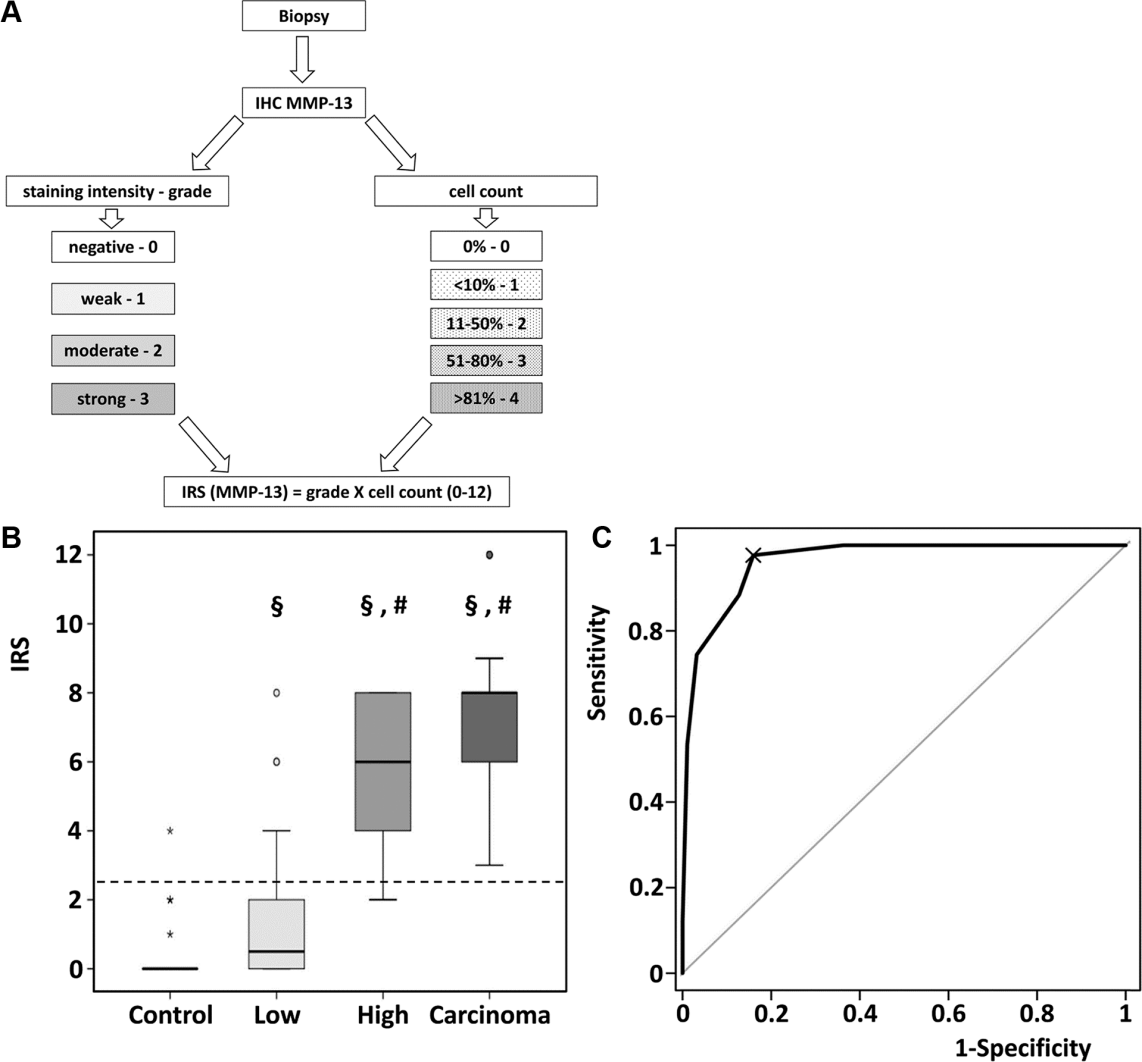
Quantitative assessment of MMP-13 by Immunoreactive Scoring (**A**) Schematic workflow for assessment of IRS. (**B**) Immunoreactive scoring of MMP-13 discriminated between low- and high grade adenoma. ^§^*p <* 0.01 in comparison to control. ^#^*p <* 0.001 in comparison to low grade adenoma. Data are presented in box-and-whisker-plots. Values deviating from the box by 1.5- to 3- fold interquartile range were defined as outliers (o). Extreme values (*) with a distance of more than 3 box-lengths were not considered for statistical analysis. (**C**) In total patient population, ROC analysis for the diagnostic accuracy of IRS for pathologic decision healthy or low grade vs. high grade adenoma or carcinoma was performed: AUC of ROC = 0.963, 95% CI 0.94–0.99, *p <* 0.001.

In low grade adenomas we detected a weak MMP-13 staining in 45% of the samples. In high grade adenomas and colorectal cancer MMP-13 was located with a moderate and strong staining. 9 (30%) of 30 (30%) of healthy controls (15 hyperplastic adenomas and 15 tumor-free surgical margins) and 32 (50%) of 64 (50%) lowgrade samples were totally negative for MMP-13, while none of the high grade adenomas and colorectal carcinoma was totally negative for MMP-13. MMP-13 expression was evaluated using a newly adopted immunoreactive score (IRS, Figure 2A) [11] increasing with the grade of dysplasia.

MMP-13 IRS increased significantly in low grade adenomas compared to healthy controls (*p* = 0.008), in high grade adenoma compared to low grade adenoma (*p* < 0.001), whereas the further increase in carcinomas did not reach statistical significance (*p* = 0.059; Figure 2B). Figure 2C shows the diagnostic value (ROC-curve) of MMP-13 IRS for discrimination between healthy and low grade adenoma vs. high grade adenoma and carcinoma with an area under the curve of 96.28% (95% CI 93.7%–98.9%, *p <* 0.001), indicating a very high diagnostic accuracy. MMP-13 IRS > 2.5 (ROC 97.67% sensitivity) comprises high grade adenoma and carcinoma. Considering the prevalence of high grade adenoma and carcinoma, the positive predictive value is 73.64%. In turn 84.04% (specificity) of biopsies are correctly identified as healthy or containing low grade adenoma. The negative predictive value is 98.75%.

MMP-13 protein expression assessed by Western blotting (Supplementary Figure S1), increased with the degree of pathological stage. In healthy tissue and low grade adenomas only weak signals could be detected, whereas in high grade adenoma and carcinoma stronger signals and bands of activated MMP-13 appeared. These findings correlate with MMP-13 IRS. β-actin loading control indicated equal loading.

Double staining of MMP-13 and cellular markers revealed a co-localization of MMP-13 with CK20 in epithelia, vWF in endothelial cells, and Vimentin in fibroblasts (Supplementary Figure S2A). No co-localisation appeared in B-cells, T-cells, macrophages and monocytes (Supplementary Figure S2B).

## DISCUSSION

The outcome of CRC depends on the extent of local and particularly metastatic tumor spread. MMPs are considered to be important in facilitating tumor invasion and spread. The expression of MMP-13 seems to be closely related to development, invasion, and progression of colorectal cancer [8, 9].

We demonstrate that MMP-13 expression, quantitatively assessed by a newly adopted IRS and verified by Western blotting, increased with pathological stage of adenoma and carcinoma development. The high diagnostic accuracy demonstrates the quality of this IRS for identification of low and high grade adenomas as well as CRC. Moreover, in high grade adenoma and cancer samples bands of activated MMP-13 point out functionalization of MMP-13 during cancerogenesis (Supplementary Figure S1). It has been shown before that high levels of MMP-13 correlate with higher rates of liver metastasis, a bad prognosis, and early relapse [9, 10]. Thus, MMP-13 IRS could also be helpful to predict metastatic behaviour, prognosis, and relapse at an early stage of cancerous and precancerous colorectal adenoma, which is definitely not accessible by conventional histology. Especially the strong increase in MMP-13 IRS from low to high grade adenoma defines an early timepoint of beneficial MMP-13 staining as early predictive cancer marker. Nevertheless, to ensure the diagnostic value of MMP-13 IRS in predicting cancer behaviour at this stage long-term follow-up studies are necessary. In previous studies we demonstrated enhanced expression of tumor-associated MMPs (MMP-2, MMP-7, MMP-9, and MMP-13) in native, unfixed, but cryo-conserved samples of patients with inflammatory bowel disease by qRT-PCR, ELISA, and immunofluorescence [6, 12]. The current study was designed to establish a translational protocol with regard to a reproducible, specific, and sensitive analysis of the tumor-associated MMP-13 in routinely assessed biopsy specimens from precancerous colorectal lesions. For the present study, histologically defined cancerous and non-cancerous colorectal adenomas but not inflammatory samples were included. Therefore, validation of MMP-13 IRS for IBD inflammatory bowel disease should be enrolled in another study.

MMP-13 is produced by fibroblasts [13], synoviocytes [14], endothelial cells [15], Kupfer cells [16], and more. We demonstrated MMP-13 expression in fibroblasts, endothelial and epithelial cells. As the amounts of MMP-13 clearly depend on the state of dysplasia, MMP-13 could be a useful marker to discriminate the different critical stages of dysplasia.

Up to now no differentiation of serrated adenomas and flat adenomas was conducted due to the limited study collective. Furthermore, subgroups were not differentiated for familiar risk factors or left and right sided lesions.

In conclusion, MMP-13 IRS represents a suitable method to assess pathologic grade of precancerous and cancerous colorectal lesions. MMP-13 has been identified as an excellent marker of high grade IEN and CRC, and may thus be applied for prognostic stratification. The identification of cellular MMP-13 sources offers a basis for targeted therapeutic modulation in CRC.

## MATERIALS AND METHODS

### Patients

The study was approved by the local ethics committee (Approval No. AZ 116/12). From June 2013 to January 2015 105 patients were enrolled for the study, the patient samples were collected by the Department of Pathology. Patients who underwent endoscopy with polypectomy or surgery had been included. A series of 137 biopsies from colorectal adenomas and colorectal cancer from 105 patients were analyzed. The biopsy material was fixed and examined concerning to hyperplastic, low or high grade dysplasia histologically as a matter of routine by two independent pathologists. Immunohistochemically staining of MMP-13 was performed and semi-quantitatively evaluated according to a novel immunoreactive score (IRS).

### Immunoreactive score (IRS)

The stained specimens were analyzed using a modified immunoreactive score adapted to Remmele et al. [11] Briefly, the staining was graded (grade: 0, negative; 1, weak; 2 moderate; 3 strong) and the percentage of positive stained cells were counted (count: 0, 0%; 1, < 10%; 2, 11–50%; 3, 51–80%; 4, > 81%). IRS is defined as grade X count and thus, runs the gamut from 0 to 12.

### Immunohistochemistry

Paraffin sections were dewaxed, unmasked by boiling in citrate buffer at pH = 6 for 10 minutes, and endogenous peroxidase activity was blocked with 3% hydrogen peroxide solution for 30 min. Protein blocker was used for 1 hour to inhibit non-specific antigen-antibody reactions (2.5 % normal horse serum; Vector Laboratories, Burlingame, USA).

Immunostaining was performed using specific antibodies for detection of MMP-13 (1:100, goat anti MMP-13 polyclonal IgG, R&D Systems, Minneapolis, USA) and suitable secondary antibodies (Impress Reagent Kit anti-goat-Ig Vector, Vector Laboratories, Burlingame, USA). For color reaction, the slides were stained with *VIP Substrate kit for peroxidase* (Vector Laboratories) and counterstained with methyl green. A violet precipitate in the cytoplasm indicates a positive reaction. Antibodies for CD3 as T-cell marker (rabbit anti CD3 polyglonal IgG, Abcam, Cambridge, UK), CD14 as monocyte marker (rabbit anti CD14 polyclonal IgG, Progen, Heidelberg, Germany), CD20 as B-cell marker (goat anti CD 20 polyclonal IgG, Santa Cruz, Dallas, USA), CD68 as marker for macrophages (mouse anti CD 68 purified IgG, Bio Legend, Fell, Germany), CK20 as colorectal epithelial marker (rabbit anti CK 20 polyclonal IgG, Protein tech, Chicago, USA), Vimentin as fibroblast marker (mouse anti Vimentin Ab-2 monoclonal IgG, Thermo Fischer, Frankfurt aM, Germany), and vWF as marker for endothelia (rabbit anti vWF polyclonal IgG, Dako, Hamburg, Germany) were used for double staining to detect cellular sources of MMP-13 expression. Secondary staining was performed with *DAB Substrate Kit for Peroxidase* (Vector Laboratories) and counterstained with methyl green. A brown reddish color was shown. Co-localization is marked by a mixed color. For negative controls equally concentrated unspecific isotype IgG were applied instead of primary antibodies.

### Protein extraction

Protein extraction from formalin-fixed paraffin embedded sections was performed as described before [17].

### Western blotting

Western blotting was performed as described elsewhere, [18] using antibodies specific for MMP-13 (Santa Cruz Biotechnology) and rabbit anti-Δ-actin (# 4970, Cell Signaling, Leiden, Netherlands) detection.

### Statistical analysis

Statistical analysis was performed with SPSS version 22 and R version 3.1.3. The IRS of hyperplastic, low grade, high grade adenomas, and colorectal carcinoma were compared by Kruskal-Wallis Test, testing whether the medians of IRS are the same in all groups. Pairwise comparison of IRS was conducted using Mann-Whitney's *U-test* and Bonferroni-Holm correction for multiple testing.

Diagnostic performance was evaluated using receiver operating characteristic (ROC) curve analysis. Confidence interval (CI) for the area under the curve (AUC) was calculated using DeLong's method. The best threshold for diagnostic purposes was determined using Youden's J statistic. ROC analyses were performed with the *pROC* R-package, version 1.8. [19].

## SUPPLEMENTARY MATERIALS FIGURES


