# Supplementary material for: Matrix metalloproteinase-13 refines pathological staging of precancerous colorectal lesions
Source: Oncotarget. 2016 Oct 4;7(45):73552–7. doi: 10.18632/oncotarget.12429 (PMC5341998; doi:10.18632/oncotarget.12429)
Supplement: Supplementary file 1 [file oncotarget-07-73552-s001.pdf]

Matrix metalloproteinase-13 refines pathological staging of precancerous colorectal lesions

Supplementary Materials

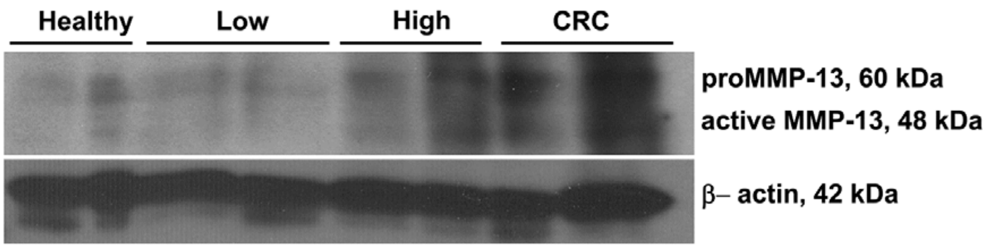

Supplementary Figure S1: MMP-13 protein expression in colon adenoma-carcinoma sequence. A representative Western blot demonstrates enhanced MMP-13 protein expression in high grade adenoma and colon carcinoma.

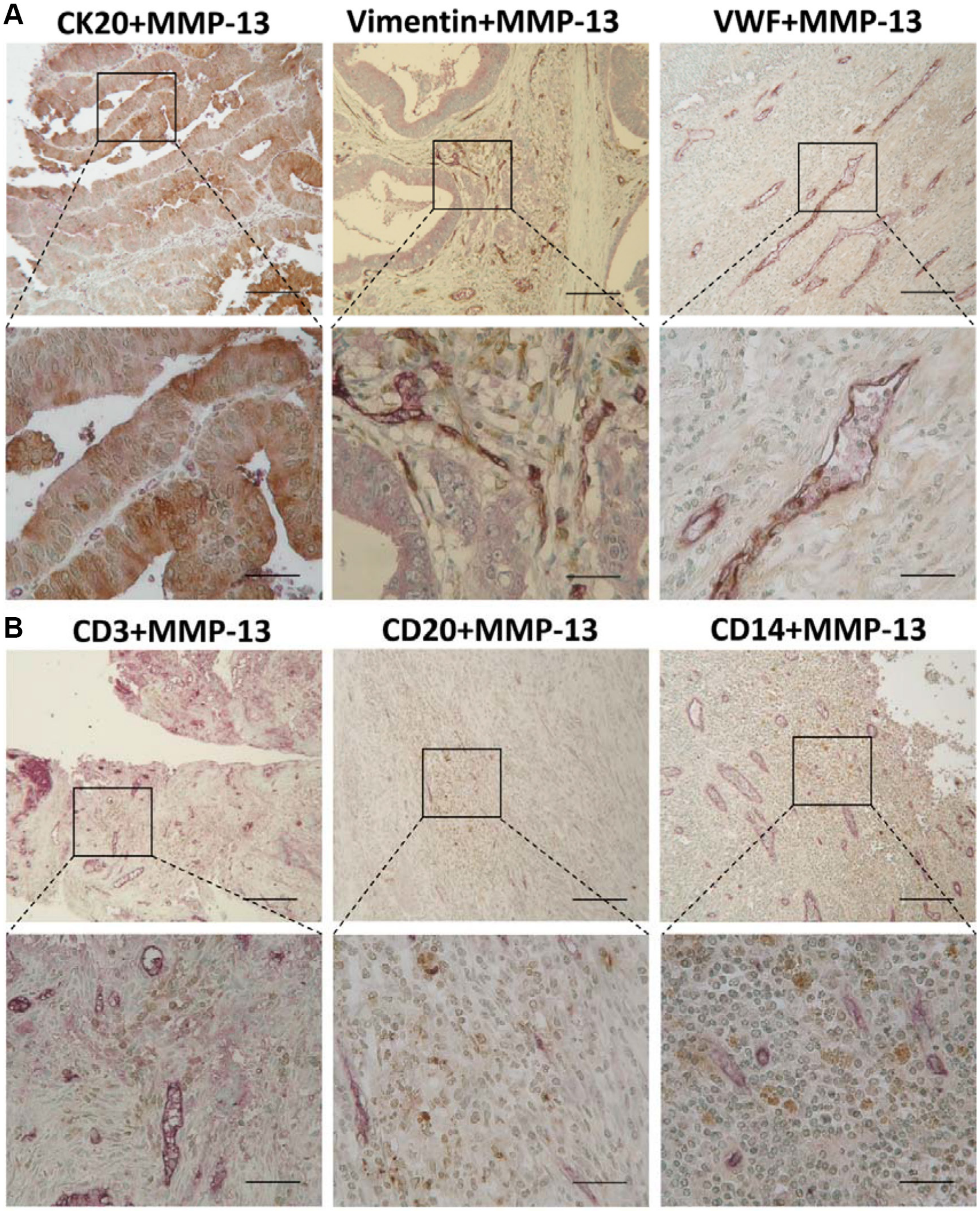

Supplementary Figure S2:Cellular sources of MMP-13 in CRC. (**A**) Immunohistochemical costainings for MMP-13 (pink) and epithelial marker CD20, fibroblast marker αSMA, and endothelial marker vWF (brown) demonstrate MMP-13 expression in epithelia, endothelia, and fibroblasts. Scale bars: 100 µm, upper panels 100× and 25 µm, lower panels 400×. (**B**) Immunohistochemical costainings for MMP-13 (pink) and T-cell marker CD3, B-cell marker CD20, and monocyte/macrophage marker CD14 (brown) in colon carcinoma. Costainings demonstrate that T-cells, B-cells, and monocytes/macrophages do not express any MMP-13. Scale bars: 100 µm, upper panels 100× and 25 µm, lower panels 400×. Representative micrographs are shown. Nuclei are stained blue.
